# Living arrangements and associations with well-being among older adults in China: a national population-based study

**DOI:** 10.1186/s12877-025-06787-8

**Published:** 2025-11-28

**Authors:** Jianfang Zhou, Yunlu Wang, Lu Yang

**Affiliations:** 1 School of Sociology and Population Studies, Nanjing University of Posts and Telecommunications, Nanjing, 210046 China; 2 School of Psychology and Public Health, La Trobe University, Melbourne, 3086 Australia; 3 Non-Communicable Diseases and Implementation Science, Baker Heart and Diabetes Institute, Melbourne, Victoria, 3000 Australia

**Keywords:** Gender, Health status, Living arrangements, Older adults

## Abstract

**Background:**

The patterns of living arrangements among older adults have changed dramatically in China from multigeneration households to more diverse forms, including a growing number of empty-nest households. However, few studies have evaluated the role of living arrangements in the health status of older adults in China. This study aims to explore the associations between living arrangements and well-being among older adults in China, with a focus on gender differences.

**Methods:**

This study used data from the 2018 China Health and Retirement Longitudinal Study (CHARLS), which is a nationally representative survey of older people, contributing to research on the health, retirement, and intergenerational support of the older population in China. A total of 6,535 participants aged 60 years or older were selected for this study. We analysed the association of living arrangements with three dimensions of health: physical health, mental health, and self-rated health among Chinese older adults using chi-square, one-way ANOVA, logistic regression, and bivariate and multivariate linear regression tests.

**Results:**

The average age of the participants was 68.35 ± 6.24 years, and 49.78% were female. The most popular living arrangement among older adults in China was living only with spouse (51.9%). Older men’s living arrangements significantly differed from older women’s (*χ*^*2*^ = 143.128, *p* < 0.001). Further, older men living alone had significantly better self-rated health than those living with their spouse and children (ß=0.273, *p* = 0.024). Among older women, those who lived alone (ß=1.557, *p* < 0.001) or only with their children (ß=1.177, *p* = 0.005) had significantly higher depression scores compared to those who lived with both their spouse and children.

**Conclusions:**

The findings of this study showed that living arrangements were significantly associated with health outcomes among older women but not older men. Older adults who did not live with spouses were more likely to have mental issues, suggesting that older adults’ well-being could be improved through health promotion programs that consider their social and living conditions.

**Supplementary Information:**

The online version contains supplementary material available at 10.1186/s12877-025-06787-8.

## Introduction

The world’s population is growing older, with China having the largest number of people aged over 60 [1]. According to data from the seventh Chinese population census in 2020, 95.37% of the older people in China lived in their own homes [2], while the average household size in China has decreased from 3.10 in 2010 to 2.62 in 2020 [3]. Under this trend of household miniaturization, which refers to the decreasing number of people living in a single household, especially for older adults in the empty-nest families, are facing challenges with both physical and mental well-being [4, 5]. It is predicted that the proportion of empty nesters, meaning the presence of only older adults in the household due to the departure of young ones, will reach 90% by 2030 of the older population [4]. The emerging population aging coexists with the transformation of family size miniaturization, has raised concerns among scholars about the ability of our families to care for older people [6, 7].

Research has suggested that living arrangements are important for older adults’ well-being. For example, older adults who moved into eldercare centers from living with family had a higher mortality rate than those who continued to live with family [8]. Moreover, older adults who lived alone were associated with a higher risk of mental illness, compared to those who lived with their partners [9]. Both the Healthy China Action (2019–2030) and the Medium and Long-term National Plan for Actively Responding to Population Ageing emphasize that there is a need to create a healthy family environment and promote a family support system to improve the health status of the older population. However, patterns of association between living arrangements and health vary substantially between countries and within countries over time due to the differences in research objectives, health indicators, and definitions of living arrangements. Some studies have demonstrated that living with family members may have better health [10–12], while others reported that functional limitation increased the probability of older adults living with their children [13]. Nevertheless, understanding older people’s living arrangements and their key role in health and well-being is of importance in implementing health promotion strategies to achieve healthy aging goals [14].

In addition, although women were reported to have a longer life expectancy than men [15, 16], research on gender differences in health showed a different story related to women’s living status. Data from Korea that included 5,046 older participants indicated that women were more likely to have depression than men when the association between living arrangements and depression was studied [10]. Studies have shown that older women were universally more vulnerable to social, economic, and health issues than older men, which may limit their choices of living arrangements and contribute to worsening health conditions [17, 18]. Literature indicated that for Chinese older women, changes in family structure may have a more significant impact on their lives and health, compared to older men [19]. For example, being widowed and living alone was related to worse health for women than for men [20]. According to the Chinese gender culture and family norms, older women were more dependent on their children, both financially and psychologically [21]. The reason is that in traditional Chinese families, women were more likely to take on household chores and child-rearing, creating a stronger mother-child bond than the bond between children and their fathers [22].

Drawing on the above literature, few studies focus on the relationship between living arrangements and the well-being status of older people in China [11]. The recent social transition and aging population have raised concerns about eldercare in China, and the family plays an intrinsic role in the care and support of older adults [23]. Different from other countries, the association between living arrangements and the health of older people in China is unclear, especially when three dimensions of health are considered. In this article, we estimate the association between living arrangements and three dimensions of health (i.e., physical health (PH), mental health (MH), and self-rated health (SRH)) among Chinese older adults and whether there are variations by gender. Understanding the role of living arrangements on health can inform advocacy efforts for personal living management, guide health education, and shape policy design and implementation.

## Methods

### Data source

The data in this study were extracted from the 2018 wave of the China Health and Retirement Longitudinal Survey (CHARLS). CHARLS was conducted as a nationally representative survey using multi-stage stratified probability proportional sampling (PPS), including data from 10,000 households and 17,500 individuals in China. The research was approved by the Ethics Review Committee of Peking University, and all respondents gave their informed consent to the investigation. This study screened the survey results of participants aged 60 years and older. Of the 17,500 individuals surveyed, 6,535 older adults were eligible for inclusion after excluding those younger than 60 years and those with missing data on key variables. Compared with the non-enrolled samples aged over 60, the age and gender distribution of the selected samples were similar, indicating that the samples in this study can still represent the overall situation of China.

## Outcome variable

The outcome variable of this study was health status. To comprehensively evaluate the health status of the older population, PH, MH, and SRH were used [24].

Physical health. Activity of Daily Living (ADL) scale was used to measure the basic activity ability of the older population [25]. The survey question was: “Do you have difficulties in dressing (bathing/showering, eating, using the toilet, urination and defecation, getting into and out of bed) because of your health and memory?“. The answers had four options: “No, I do not have any difficulty; I have difficulty but can still do it; Yes, I have difficulty and need help; I cannot do it “. If the respondent selected “Yes, I have difficulty and need help” or “I cannot do it”, the corresponding item was re-labeled as “disabled”. Then the study divided the samples into four categories according to the number of disabled items: fully self-care (0 items “disabled”), mild disability (1–2 items “disabled”), moderate disability (3–4 items “disabled”) and severe disability (5–6 items “disabled”).

Mental health. Mental health was measured with the Center for Epidemiological Studies Depression Scale (CES-D 10) [26]. There were ten items in total, and the answer options were “rarely or none of the time (< 1 day); some or a little of the time (1–2 days); Occasionally or a moderate amount of the time (3–4 days); most or all of the time (5–7 days); do not know; refuse to answer”. For the eight positive items, response anchors range from 0 to 3. After reverse-coding two negative items (hopeful and happy), the total score range was 0 ~ 30 points. The higher the score, the heavier the tendency to depression and the unhealthier the psychological status.

Self-rated health (SRH). SRH is widely used in population health studies and is considered a good predictor of morbidity and mortality [27]. The question “Would you say your health is very good, good, fair, poor, or very poor? “, was used to assess SRH. SRH is a 5-point variable, scored from 1 (very poor) to 5 (very good).

## Independent variable

Living arrangements. The study used two questions to divide the living arrangement into four categories (“Living alone; Living with spouse only; Living with children only; Living with both children and spouse”) [28]. The first question used was “What is your marital status?” to find out whether those living with spouses or not, with six options for the answer: married with spouse present; married but not living with spouse temporarily for reasons such as work; separated; divorced; widowed; never married. If the respondent selected “married with spouse present”, it is considered as “living with spouse”, otherwise, it is considered as “not living with your spouse”. Another question used was “Where does [XChildName] live?“, to find out whether those were living with a child(ren) or not. The answers were: living with [Name of the family respondent] and not financially independent; living with [Name of the family respondent] and financially independent; living in houses in the same/neighborhood courtyard (s) or flats in the same/neighboring; other County/district-village/community; living abroad. If the children of the respondent have any of the first three answers, the respondent is considered to be “living with their children”; otherwise, it is considered “not living with children”.

## Covariates

Based on existing research findings [29, 30], covariates were set to reduce multicollinearity [31]. Personal characteristics include age, education level, and work status. The marital status was not included in this study because it was closely related to the living arrangements, and the living arrangements information covered the marriage information of most participants. Regional characteristics include regional economic types and areas of residence. The coding for all variables can be found in the Supplementary Table S1.

### Statistical analysis

Measures of frequency and percentage were used for categorical variables, and the chi-square test (*χ*^*2*^ test) was used to compare between groups. For continuous variables that follow a normal distribution, the mean$(\overset-x\pm s)$ was used. One-way analysis of variance (One-Way ANOVA) was used to compare the differences in four living arrangement groups. We employed ordered logistic regression to explore the association between living arrangements and PH and SRH outcomes (ordinal variables) [32]. For the mental health outcome of depression (binary variable), due to its non-normal distribution, a generalized linear model was used to explore the association between living arrangements and mental health [33]. Separate regression models for females and males were used to investigate gender differences, as covariate status (such as age, education) differed by gender, and may have gender-specific associations with health outcomes [34, 35]. Robustness checks were conducted to assess robustness by removing those aged over 80, who represented less than 8.00% of male and female samples. A *p*-value less than 0.05 (*p* < 0.05) is considered statistically significant. All statistical analyses were conducted with SPSS 25.0.

### Result

#### Basic characteristics

Personal characteristics (Table 1). This study comprised 3,282 men (50.22%) and 3,253 women (49.78%), with a mean age of 68.35 ± 6.24 years. The average age for the male and female participants were 68.64 ± 6.32 years and 68.06 ± 6.14 years, respectively. The educational level of the participants was generally low. There were less than 40% of the older participants had undergraduate degrees or higher. Over half of the participants were employed, with a higher percentage of older men (58.74%) continuing to work compared to older women (47.19%).

**Table 1:** Characteristics of participants between older men and women

Characteristics	Values	Older men (*n* = 3282)	Older women (*n* = 3253)	
Age	---	68.64±6.32	68.06±6.14	***F*** **= **14.273 ***p*** < 0.001
Education	Illiterate	9(0.27%)	9(0.28%)	*χ*^*2*^ = 2. 266 *p* = 0.559
	Primary school and lower	460(14.02%)	468(14.39%)	
	Middle school	1818(55.39%)	1845(56.72%)	
	College and above	995(30.32%)	931(28.62%)	
Working (including farming)	No	1354(41.26%)	1718(52.81%)	***χ***^***2***^ = 87.603 ***p*** < 0.001
	Yes	1928(58.74%)	1535(47.19%)	
Regional types	East	1005(30.62%)	980(30.13%)	*χ*^*2*^ = 4.303 *p* = 0.231
	Middle	965(29.40%)	920(28.25%)	
	West	1069(32.57%)	1070(32.89%)	
	North-east	243(7.40%)	283(8.70%)	
Area of residence	Rural	1966(59.90%)	1838(56.50%)	***χ***^***2***^ = 7.767 ***p*** = 0.003
	Urban	2731(40.10%)	1415(43.50%)	

Regional characteristics (Table 1). Of all the participants, 30.37% were from the Eastern region, 28.84% were from the middle region, 32.73% from the Western region, and 8.05% were from the Northeastern region of China. The proportion of participants residing in rural areas was 58.21%. Additionally, it was observed that a greater proportion of older men resided in rural areas (59.90%).

## Living arrangements and health status

The proportions of older participants who lived alone, lived only with spouse, lived only with children, and lived with both spouse and children were 12.35%, 51.91%, 9.67%, and 26.07%, respectively. Chi-square test showed a significant difference in living arrangements between older men and older women (*χ*^*2*^ = 143.128, *p* < 0.001). Older women were more likely to live alone and live only with their children (Table 2).

**Table 2:** Living arrangements and health status of the older population between male and female

Variable	Values	Older men (*n* = 3282)	Percent	Older women (*n* = 3253)	Percent	*χ* ^2^	*p*
		Cases		Cases			
Living arrangements	Live alone	328	9.99	479	14.72	143.128	< 0.001
	Live only with spouse	1827	55.67	1565	48.11		
	Live only with children	202	6.15	430	13.22		
	Live with both spouse and children	925	28.18	779	23.95		
ADL	Fully self-care	3057	93.14	2896	89.03	36.645	< 0.001
	Mild disability	185	5.64	306	9.41		
	Moderate disability	27	0.82	37	1.14		
	Severe disability	13	0.40	14	0.43		
SRH	Very poor	235	7.16	237	7.29	26.223	< 0.001
	Poor	655	19.96	796	24.47		
	Fair	1623	49.45	1581	48.60		
	Good	391	11.91	330	10.14		
	Very good	378	11.52	309	9.50		

Most of the participants (*N* = 5,953, 91.09%) could take care of themselves. A significant difference was observed between older men and older women in terms of their ADL (*χ*^*2*^ = 36.645, *p* < 0.001), with a higher proportion of women experiencing disabilities. The depression scores of the older men and the older women were 7.60±6.12 and 9.80±7.12, respectively. There was a significant difference in MH between the two genders (*F* = 178.991, *p* < 0.001). Almost half of the participants (49.03%) rated their health as “fair”, with 29.42% reporting poor or very poor health, and 21.54% reporting good or very good health. A significant difference was found in the self-rate of health status between older men and older women (*χ*^*2*^ = 26.223, *p* < 0.001), with a higher proportion of poor SRH among older women.

### Comparison of health status between older men and women with different living arrangements

There was no significant difference in the ADL of older men across different living arrangements (Table 3). However, there was a significant difference in the ADL of older women across different living arrangements (*χ*^*2*^ = 21.116, *p* = 0.012). Table 4 reports a significant correlation between living arrangements and depression scores for both older men and older women (*p* < 0.05). The depression scores of older men and older women living alone or only with their children were significantly higher than those of individuals in the other two types of living arrangements. Table 5 shows that no significant differences were observed in the SRH of older men across different living arrangements. However, there was a significant difference in the SRH of older women across different living arrangements (*χ*^*2*^ = 25.455, *p* = 0.013). There was a higher proportion of older women living with both spouse and children who reported fair self-rated health.

**Table 3:** Comparison of ADL between older men and women with different living arrangements [n (%)]

Gender	Living arrangements	ADL				
		Fully self-care	Mild disability	Moderate disability	Severe disability	*χ*^*2*^, *p*
Male	Live alone	307(93.60)	18(5.49)	1(0.30)	2(0.61)	6.442, 0.695
	Live only with spouse	1704(93.27)	102(5.58)	14(0.77)	7(0.38)	
	Live only with children	181(89.60)	17(8.42)	3(1.49)	1(0.50)	
	Live with both spouse and children	865(93.51)	48(5.19)	9(0.97)	3(0.32)	
Female	Live alone	418(87.27)	57(11.90)	3(0.63)	1(0.21)	21.116, 0.012
	Live only with spouse	1406(89.84)	140(8.95)	14(0.89)	5(0.32)	
	Live only with children	368(85.58)	47(10.93)	10(2.33)	5(1.16)	
	Live with both spouse and children	704(90.37)	62(7.96)	10(1.28)	3(0.39)	

**Table 4:** Comparison of mental health between older men and women with different living arrangements

Living arrangements	Older Men				Older Women		
	Cases	Mean	Standard deviation	Cases		Mean	Standard deviation
Live alone	328	8.41	6.30	479		10.87	7.51
Live only with spouse	1827	7.32	6.01	1565		9.60	7.06
Live only with children	202	8.49	6.48	430		10.34	7.36
Live with both spouse and children	925	7.68	6.15	779		9.24	6.75
Model parameters	F = 4.697, *P* = 0.003				F = 6.503, *P* < 0.001		

**Table 5:** Comparison of self-rated health between older men and women with different living arrangements [n (%)]

Gender	Living arrangements	Self-rated health					
		Very poor	Poor	Fair	Good	Very good	χ^2^, P
Men	Live alone	22(6.71)	69(20.73)	155(47.26)	40(12.20)	43(13.11)	9.279, 0.679
	Live only with spouse	135(7.39)	355(19.43)	892(48.82)	234(12.81)	211(11.55)	
	Live only with children	16(7.92)	46(22.77)	95(47.03)	25(12.38)	20(9.90)	
	Live with both spouse and children	62(6.70)	186(20.11)	481(52.00)	92(9.95)	104(11.24)	
Women	Live alone	43(8.98)	124(25.89)	214(44.68)	59(12.32)	39(8.14)	25.475, 0.013
	Live only with spouse	112(7.16)	386(24.66)	764(48.82)	154(9.84)	149(9.52)	
	Live only with children	38(8.84)	124(28.84)	192(44.65)	39(9.07)	37(8.60)	
	Live with both spouse and children	44(5.65)	162(20.80)	411(52.76)	78(10.01)	84(10.78)	

### Multivariate analysis of the influence of living arrangements on the health of the older population of different genders

After controlling for individual characteristics and regional factors (covariates), the influence of living arrangements on the PH, MH, and SRH between older men and older women is presented in Tables 6, 7 and 8. Regarding PH (ADL), the association with living arrangements was not significant for either older men and women (Table 6). Regarding MH, among older women, those who lived alone [ß (95% CI) = 1.557 (0.753, 2.361)] or only with their children [ß (95% CI) = 1.177 (0.353, 2.000)] had significantly higher depression scores compared to those who lived with both their spouses and children (Table 7). Table 8 showed that older men living alone had significantly better SRH compared to those living with their spouses and children (ß=0.273, 95%CI: 0.035, 0.511, *p* = 0.024). On the other hand, the SRH of older women who lived only with their spouses (ß=−0.205, 95%CI: −0.366, −0.044, *p* = 0.013) or only with children (ß=−0.284, 95%CI: −0.509, −0.058, *p* = 0.014) was significantly worse than that of those living with both their spouses and children. The robustness checks’ results (Tables 9, 10 and 11) were consistent with the main findings.

**Table 6:** Multivariate ordinal logistic regression analysis of the association of living arrangements with ADL between older men and women

	Standard Error	Wald	*P*	ß (95%CI)	
Men (living with both spouse and children as reference group)					
Live alone	0.275	2.638	0.104	−0.447(−0.987,0.092)	
Live only with spouse	0.168	0.236	0.627	−0.082(−0.411,0.248)	
Live only with children	0.282	0.059	0.809	−0.068(−0.621,0.484)	
Model parameters	*R*^2^ = 0.107 (when living arrangement is not included), *R*^2^ = 0.109, χ^2^ = 160.507, *P* < 0.001				
Women (living with both spouse and children as reference group)					
Live alone	0.198	2.388	0.122	−0.306(−0.694,0.082)	
Live only with spouse	0.152	0.017	0.895	−0.020(−0.317,0.277)	
Live only with children	0.195	0.073	0.786	−0.053(−0.436,0.330)	
Model parameters	*R*^2^ = 0.099 (when living arrangement is not included), *R*^2^ = 0.101, χ^2^ = 186.164, *P* < 0.001				

covariates: age, education level, work status, regional economic types, and areas of residence

**Table 7:** Analysis of the general linear regression model of the influence of living arrangements on mental health between older men and women

	Wald χ^2^	Standard Error	*P*	ß(95%CI)		
Men (living with both spouse and children as reference group)						
Live alone	0.433	0.384	0.510	0.253(−0.500,1.005)		
Live only with spouse	2.499	0.239	0.114	−0.378(−0.847,0.091)		
Live only with children	0.642	0.465	0.423	0.373(−0.539,1.285)		
Model parameters	Standard Person χ^2^ = 3282.000, likelihood ratio χ^2^ = 245.724, *P* < 0.001					
Women (living with both spouse and children as reference group)						
Live alone	14.394	0.410	< 0.001	1.557(0.753,2.361)		
Live only with spouse	3.231	0.300	0.072	0.540(1.128,3.231)		
Live only with children	7.836	0.420	0.005	1.177(0.353,2.000)		
Model parameters	Standard Person χ^2^ = 3253.000, likelihood ratio χ^2^ = 276.833, *P* < 0.001					

covariates: age, education level, work status, regional economic types, and areas of residence

**Table 8:** Ordinal logistic regression analysis of the association of living arrangements with self-rated health between older men and women

	Standard Error	Wald	*P*	ß (95%CI)	
Men (living with both spouse and children as reference group)					
Live alone	0.121	5.063	0.024	0.273(0.035,0.511)	
Live only with spouse	0.076	1.782	0.182	0.101(−0.047,0.249)	
Live only with children	0.147	0.658	0.417	0.119(−0.169,0.407)	
Model parameters	*R*^2^ = 0.057 (when living arrangement is not included), *R*^2^ = 0.058, χ^2^ = 184.222, *P* < 0.001				
Women (living with both spouse and children as reference group)					
Live alone	0.112	1.702	0.192	−0.146(−0.366,0.074)	
Live only with spouse	0.082	6.201	0.013	−0.205(−0.366, −0.044)	
Live only with children	0.115	6.087	0.014	−0.284(−0.509, −0.058)	
Model parameters	*R*^2^ = 0.052 (when living arrangement is not included), *R*^2^ = 0.054, χ^2^ = 168.433, *P* < 0.001				

covariates: age, education level, work status, regional economic types, and areas of residence

**Table 9:** Multivariate ordinal logistic regression analysis of the association of living arrangements with ADL between older men and women

	Standard Error	Wald	*P*	ß (95%CI)	
Men (living with both spouses and children as reference group)					
Live alone	0.335	3.784	0.052	−0.651(−1.308,0.005)	
Live only with spouse	0.179	0.001	0.989	−0.003(−0.354,0.349)	
Live only with children	0.337	0.001	0.992	−0.003(−0.665,0.658)	
Model parameters	*R*^2^ = 0.109, χ^2^ = 139.398, *P* < 0.001				
Women (living with both spouses and children as reference group)					
Live alone	0.209	0.813	0.367	−0.189(−0.5994,0222)	
Live only with spouse	0.156	0.017	0.896	−0.020(−0.325,0.285)	
Live only with children	0.209	0.158	0.691	−0.083(−0.493,0.327)	
Model parameters	*R*^2^ = 0.087, χ^2^ = 144.064, *P* < 0.001				

covariates: age, education level, work status, regional economic types, and areas of residence

**Table 10:** Analysis of the general linear regression model of the influence of living arrangements on mental health between older men and women

	Wald χ^2^	Standard Error	*P*	ß(95%CI)		
Men (living with both spouse and children as reference group)						
Live alone	0.433	0.384	0.510	0.253(−0.500,1.005)		
Live only with spouse	2.499	0.239	0.114	−0.378(−0.847,0.091)		
Live only with children	0.642	0.465	0.423	0.373(−0.539,1.285)		
Model parameters	Standard Person χ^2^ = 3050.000, likelihood ratio χ^2^ = 237.862, *P* < 0.001					
Women (living with both spouse and children as reference group)						
Live alone	13.602	0.428	< 0.001	1.577(0.739,2.416)		
Live only with spouse	2.824	0.303	0.093	0.509(1.103,2.824)		
Live only with children	10.527	0.434	0.001	1.410(0.558,2.261)		
Model parameters	Standard Person χ^2^ = 3067.000, likelihood ratio χ^2^ = 267.744, *P* < 0.001					

covariates: age, education level, work status, regional economic types, and areas of residence

**Table 11:** Ordinal logistic regression analysis of the association of living arrangements with self-rated health between older men and women

	Standard Error	Wald	*P*	ß (95%CI)	
Men (living with both spouse and children as reference group)					
Live alone	0.127	4.808	0.028	0.273(0.030,0.528)	
Live only with spouse	0.077	1.778	0.182	0.103(−0.048,0.255)	
Live only with children	0.162	0.276	0.599	0.085(−0.233,0.403)	
Model parameters	*R*^2^ = 0.058, χ^2^ = 170.899, *P* < 0.001				
Women (living with both spouse and children as reference group)					
Live alone	0.117	1.488	0.223	−0.143(−0.373,0.087)	
Live only with spouse	0.083	5.938	0.015	−0.203(−0.366, −0.040)	
Live only with children	0.119	8.081	0.004	−0.338(−0.572, −0.105)	
Model parameters	*R*^2^ = 0.055, χ^2^ = 162.230, *P* < 0.001				

covariates: age, education level, work status, regional economic types, and areas of residence

## Discussion

To our knowledge, this is the first study to examine the living arrangements associated with three dimensions of health among older adults in China. The findings underscore the complexity of gender, highlighting the role of various types of living arrangements in physical health, mental health, and self-rated health among the older population. The main findings are: (1) gender inequality in the health of older adults; (2) older people who lived alone or lived only with children were more vulnerable to mental health issues; (3) significant associations between living arrangements and health were observed among older women but not among older men. This study shows that gender inequality in the health of older adults was evident, with older women experiencing worse health outcomes compared to older men. This finding is in accordance with other studies conducted in China [36], Korea [37], India [38], and the US [39]. Similarly, a study comprising 19,841 older Chinese from 2011 to 2015 showed that women were 1.31 times more likely to have multimorbidity compared to men [40]. This disparity of health status between genders may be attributed to lower personal health capital and social capital among older women compared to older men, including socioeconomic status, health behaviors, and immune-system responses [41, 42]. Therefore, it is crucial to adopt a gender perspective when striving to improve healthy aging, such as providing health support (health education and health counseling programs) to older women and their family members and promoting the extension of healthy life expectancy are important measures to address gender inequality in health.

This study showed that both older men and women had significantly higher average depression scores when their living arrangements were “living alone” or “living with children only,” compared to the other two living arrangements (living with a spouse and living with both spouses and children). The finding is consistent with a study comprising 22,105 middle-aged and older Canadian adults, indicating that people without partners had lower levels of MH and higher levels of loneliness than those with partners [43]. Similarly, data from the US National Health Interview survey of 76,277 adults over 65 reported that those living with a spouse were less likely to have multimorbidity than those living with children [44]. These results suggest that having a spouse living together is an important protective factor against depression among the older population [10]. This may be attributed to long-term interactions between older couples, which form strong emotional bonds in today’s Chinese families [45]. Given these findings, tailored social-network interventions for widowed older adults who live alone could be beneficial in improving health outcomes [46].

This research showed that living arrangements were significantly associated with health outcomes among older women but not older men. In the context of China’s filial piety, meaning that parents have historically lived with their adult children due to the obligation of adult children, the finding that older women living only with their children had relatively worse MH is interesting [13]. This is in accordance with other studies suggesting that older women were vulnerable to declines in their standard of living [47], household income [18, 48], increased risks of poverty [49] and loss of home ownership [50] following adverse life events. The reason can be supported by the role-strain theory, in which older women living with children have greater caregiving burdens compared to older men, such as caring for their grandchildren [51, 52], which may be detrimental to mental health [53]. Moreover, compared to older men, older women in China generally receive lower pensions [54], which makes them more dependent on their children in later life, thus increasing vulnerability to mental health problems. Our study also showed that older women who lived alone had poorer mental health, which may be due to the fact that they were more likely to experience social isolation [55]. This finding is consistent with social support theory, indicating that stronger social connections are associated with reduced risk of mental disorder in women [56, 57]. Future research is needed to examine how changes in living arrangements affect older women’s health, and how to assist them in actively adapting to such changes. Interventions or social support programs to help older women adjust to new living arrangements could be helpful in their health management.

This research also found that older men living alone had better SRH than those living with their spouses and children. This finding is in accordance with another study, indicating that adults over 65 living alone had better health than those living with others [58]. However, the correlation between living alone and SRH did not extend to mental health and physical health outcomes in this study. This might be related to the fact that self-rated health is an individual’s subjective assessment of their own health status, integrating biological, social and functional aspects of a person, not all of which can be captured by objective outcomes such as ADL and mental health [59]. Alternative explanations, such as reverse causation and survivor effect, need to be considered. For example, older men living alone may have lower health expectations due to loneliness [60]. Also, healthy older men tend to live on their own, which relates to a preference for independence and freedom [61]. In this case, better health is a prerequisite for living alone, rather than an outcome of living alone. In addition, there are studies suggested that older men who lived alone were more likely to be healthy, while those with poorer health may be more likely to live with their families to receive care [62, 63]. The contradiction between poorer mental health among older women living alone and better self-rated health among older men living alone can be partly explained by gender differences in social roles in China. In traditional Chinese society, older women tend to rely more on adult children for financial support, compared to older men, thus experiencing greater social isolation when living alone [64]. On the other hand, older men may have more economic resources than older women, allowing them to maintain social connections outside the household, thus having better health when living alone [57]. Further research should explore the underlying factors influencing older men’s subjective health status, especially when they live alone.

This study showed no significant difference in health among the older population living in empty nest households, compared to other living arrangements. This result differs from studies conducted in South Korea [10] but aligns with another study conducted in China [65]. A study in China showed that empty nesters had higher life expectancy compared to non-empty nesters across all age groups [66]. These findings suggest that the older population in China may possess a certain adaptability to the empty nest living arrangement, and their health status may not be worse off than those living with children. The traditional family mode of living with children did not demonstrate clear advantages for the health of older people. The ongoing trend of family miniaturization and the prevalence of empty nests may not have a negative impact on the health of the older population, which requires further research.

This study has some limitations. First, because this is a cross-sectional study, causality cannot be established. Second, the use of secondary analysis of existing survey data limited the availability of indicators for analysis, particularly in examining family relationships related to living arrangements. Third, although CESD-10 is a reliable and valid measure of depression, it is not a clinical diagnostic instrument and therefore may misclassify some respondents’ depressive status. Last, the study did not explain why living arrangements affect health among older people, which is important to delve into the underlying mechanism so that a targeted intervention can be implemented. Despite the limitations of this study, the highlight of this study is that it represents the first and largest investigation of its kind. It examines the association of living arrangements with well-being, from a gender perspective, using a nationwide representative sample in China. Therefore, this study’s findings are generalizable to the entire country, providing insights into the overall well-being of the older population in China. Moreover, the practical implication of this study is the need to provide tailored support to older women, who are more vulnerable in terms of health and well-being in later life, with a focus on their living arrangements. For example, implementing community support programs tailored for older women who live alone and addressing social isolation and loneliness could contribute to their risk of decreased health status.

## Conclusion

This research identified significant associations between living arrangements and well-being among older women, but not among older men. Older adults who did not live with spouses were more likely to have mental issues, suggesting that older adults’ well-being could be facilitated through health promotion programs that consider their social and living conditions.

## Supplementary Information

Supplementary Material 1

## Data Availability

No datasets were generated or analysed during the current study.

## Abbreviations

ADL: Activity of daily living
CES-D: Center for epidemiological studies depression scale
CHARLS: China health and retirement longitudinal study
MH: Mental health
PPS: Probability proportional sampling
PH: Physical health
SRH: Self-rated health
