# Supplementary material for: Living arrangements and associations with well-being among older adults in China: a national population-based study
Source: BMC Geriatr. 2025 Nov 28;25:1071. doi: 10.1186/s12877-025-06787-8 (PMC12751397; doi:10.1186/s12877-025-06787-8)
Supplement: Supplementary file 1 — Supplementary Material 1 [file 12877_2025_6787_MOESM1_ESM.docx]

| **Supplementary File 1. Coding information** | | | |
| --- | --- | --- | --- |
| **Variable** | **Original CHARLS question(s) and response options** | **Coding procedure** | **Final coding used in analysis** |
| **Independent variable** | | | |
| **Living arrangements** | BE001 What is your marital status? (1. Married and live with spouse; 2. Married but don’t live with spouse temporarily for reasons such as work; 3. Separated, don’t live together as a couple anymore; 4. Divorced; 5. Widowed; 6. Never married)  CB053_i Where does [X Child Name[i] live? (1. living with [Name of the family respondent] and not financially independent; 2. living with [Name of the family respondent] and financially independent; 3. living in houses in the same/neighborhood courtyard (s) or flats in the same/neighboring; 4. other County/district-village/community; living abroad) | For BE001, if selecting answer 1, the respondent is considered to be “living with their spouse, otherwise, it is considered “not living with spouse”.  For CB053_i, if the children of the respondent have any of the first three answers, the respondent is considered to be “living with their children”; otherwise, it is considered “not living with children”. | Live alone=0  Live only with spouse=1  Live only with children=2  Live with both spouse and children=3 |
| **Outcome variables** | | | |
| **Physical health (ADL)** | DB010-DB015 Do you have difficulties with dressing (bathing/showering, eating, eating, using the toilet, urination and defecation, getting into and out of bed) because of your health and memory? (1. No, I do not have any difficulty; 2. I have difficulty but can still do it; 3. Yes, I have difficulty and need help; 4. I cannot do it) | If the respondent selected 3 or 4, then they were labelled as “disabled”. According to the number of disabled items, categorize: fully self-care (0 items disabled), mild disability (1-2 items disabled), moderate disability (3-4 items disabled) and severe disability (5-6 items disabled) | Fully self-care=0  Mild disability=1  Moderate disability=2  Severe disability=3 |
| **Mental Health (CES-D 10)** | DC009 I was bothered by things that don’t usually bother me;  DC010 I had trouble keeping my mind on what I was doing;  DC011 I felt depressed;  DC012 I felt everything I did was an effort;  DC013 I felt hopeful about the future;  DC014 I felt fearful;  DC015 My sleep was restless;  DC016 I was happy;  DC017 I felt lonely;  DC018 I could not get “going”. | For the eight positive items: rarely or none of the time (<1 day)=0; some or a little of the time (1-2 days)=1; Occasionally or a moderate amount of the time (3-4 days)=2; most or all of the time (5-7 days)=3.  For item DC012 and DC016, reverse coding:  rarely or none of the time (<1 day)=3; some or a little of the time (1-2 days)=2; Occasionally or a moderate amount of the time (3-4 days)=1; most or all of the time (5-7 days)=0. | The total score range was 0~30 points. |
| **Self-rated health (SRH)** | DA002 Would you say your health is very good, good, fair, poor or very poor? | The value is assigned according to the answer chosen by the respondent. | Very poor=0  Poor=1  Fair=2  Good=3  Very good=4 |
| **Covariates** | | | |
| **Sex** | BA000_W2_3 The Interviewer records the Respondent’s gender | The value is assigned according to the answer chosen by the respondent. | Female=0  Male=1 |
| **Age** | BA002 What’s your actual date of birth? (BA002_1) Year | Survey year 2018- birth year | Continuous variable |
| **Education** | BD001_W2_4 What’s the highest level of education you have now (not including adult education)? (1. No formal education (illiterate); 2.Did not finish primary school; 3. Sishu/home school; 4. Elementary school; 5. Middle school; 6. High school; 7. Vocational school; 8. Two-/Three-Year College/Associate degree; 9. Four-Year College/Bachelor’s degree; 10. Master’s degree; 11. Doctoral degree/Ph.D.) | Merging 2, 3, 4 as “primary school and lower”; Merging 5, 6, 7 as “middle school”; Merging 8, 9,10, 11 as “College degree and above”. | Illiteracy=0  Primary school and lower=1  Middle school=2  College degree and above=3 |
| **Working**  **(including farming)** | FC008 Did you engage in agricultural work for at least 10 days in the past year for your own household?  FC001 Did you work for other farmers/employers and get paid for at least ten days in the past year?  FA002_W4 Not including agricultural work, did you work for at least one hour last week in paid work, individual business or family business without getting paid?  FA005 Do you expect to get back to this job at a definite time in the future or within 6 months? | If the answer for any of these questions was “yes”, them calculated as “yes” for working, otherwise as “no” | No=1  Yes=2 |
| **Regional types** | ID code | Province information can be identified using the ID code. Then, categorize into eastern, central, western, and northeastern regions. | East=1  Middle=2  West=3  Northeast=4 |
| **Area of residence** | Community ID | Areas of residence are classified according to community ID coding rules. | Rural=1  Urban=2 |
